# Ikaros-Associated Diseases: From Mice to Humans and Back Again

**DOI:** 10.3389/fped.2021.705497

**Published:** 2021-07-16

**Authors:** Brigette Boast, Cristiane de Jesus Nunes-Santos, Hye Sun Kuehn, Sergio D. Rosenzweig

**Affiliations:** ^1^Department of Immunology and Infectious Disease, John Curtin School of Medical Research, The Australian National University, Canberra, ACT, Australia; ^2^Immunology Service, Department of Laboratory Medicine, National Institutes of Health Clinical Center, Bethesda, MD, United States

**Keywords:** primary immunodeficiency, inborn errors of immunity, transcription factors, infection, cytopenia, T cell, B cell, hypogammaglobulinemia

## Abstract

The normal expression of Ikaros (IKZF1) is important for the proper functioning of both the human and murine immune systems. Whilst our understanding of IKZF1 in the immune system has been greatly enhanced by the study of mice carrying mutations in *Ikzf1*, analyses of human patients carrying germline *IKZF1* mutations have been instrumental in understanding its biological role within the human immune system and its effect on human disease. A myriad of different mutations in *IKZF1* have been identified, spanning across the entire gene causing differential clinical outcomes in patients including immunodeficiency, immune dysregulation, and cancer. The majority of mutations in humans leading to IKAROS-associated diseases are single amino acid heterozygous substitutions that affect the overall function of the protein. The majority of mutations studied in mice however, affect the expression of the protein rather than its function. Murine studies would suggest that the complete absence of IKZF1 expression leads to severe and sometimes catastrophic outcomes, yet these extreme phenotypes are not commonly observed in patients carrying *IKZF1* heterozygous mutations. It is unknown whether this discrepancy is simply due to differences in zygosity, the role and regulation of IKZF1 in the murine and human immune systems, or simply due to a lack of similar controls across both groups. This review will focus its analysis on the current literature surrounding what is known about germline IKZF1 defects in both the human and the murine immune systems, and whether existing mice models are indeed accurate tools to study the effects of IKZF1-associated diseases.

## Introduction

### IKZF1 and Lymphocyte Lineage Commitment

*IKZF1* (Ikaros Zinc Finger Protein 1), encoding for Ikaros, is a zinc finger transcription factor that is essential for immune cell development, homeostasis, and function (1). It regulates transcriptional programs through the coordination of six highly conserved C2H2 zinc finger (ZF) domains; the first four at the N-terminus are essential for regulating gene transcription through DNA binding, and the last two at the C-terminus facilitate multimer formation as both a homodimer and as a heterodimer with other family members: IKZF2 (Helios), IKZF3 (Aiolos), IKZF4 (Eos), and IKZF5 (Pegasus) (2). IKZF1 is able to regulate gene expression through direct binding and activating of promoter regions (2–4), but predominantly negatively regulates gene transcription through repression of chromatin and recruitment of co-repressors (4–11). The expression of IKZF1 is important in guiding hematopoietic cell fate decisions in the early development of uncommitted lymphocytes as well as controlling transcriptional programs essential for later B and T cell development. IKZF1 expression can be detected from the earliest stage of hematopoietic development in the fetal liver and adult bone marrow and its expression is continued through until mature B and T cells (12–15).

Lymphocyte development first begins with pluripotent and self-renewing hematopoietic stem cells (HSCs) upregulating *Flt3* to become multipotent progenitors (MPPs) (16). This upregulation of *Flt3* removes the ability of MPPs to self-renew and restricts them to either a lymphocyte or myeloid fate (17). In mice, the subsequent upregulation of interleukin-7 receptor (IL7Rα or CD127) will commit a cell to the lymphoid lineage, defining them as a common lymphoid progenitor (CLP) (18), thereby facilitating a dependency on IL7 that is secreted by stromal cells in the bone marrow (19–21). This however, is true only in mice as human B cells do not require IL7 for normal development (22). From here, CLPs can then differentiate into either B, T, dendritic cells, or natural killer cells (18, 23, 24). IKZF1 is an important regulator that guides lymphoid commitment. In the complete absence of IKZF1 expression, a failure to establish the lymphoid program in the fetal liver results in the absence of B and T cell development (25, 26) that can be attributed to the loss of *Il7r* and *Flt3* upregulation (27). The expression of IKZF1 however, is not limited to early hematopoietic progenitors as IKZF1 also has important roles both early in B cell development (28) and in the regulation of mature B cells (29).

### IKZF1 Isoforms

**Figure 1:**
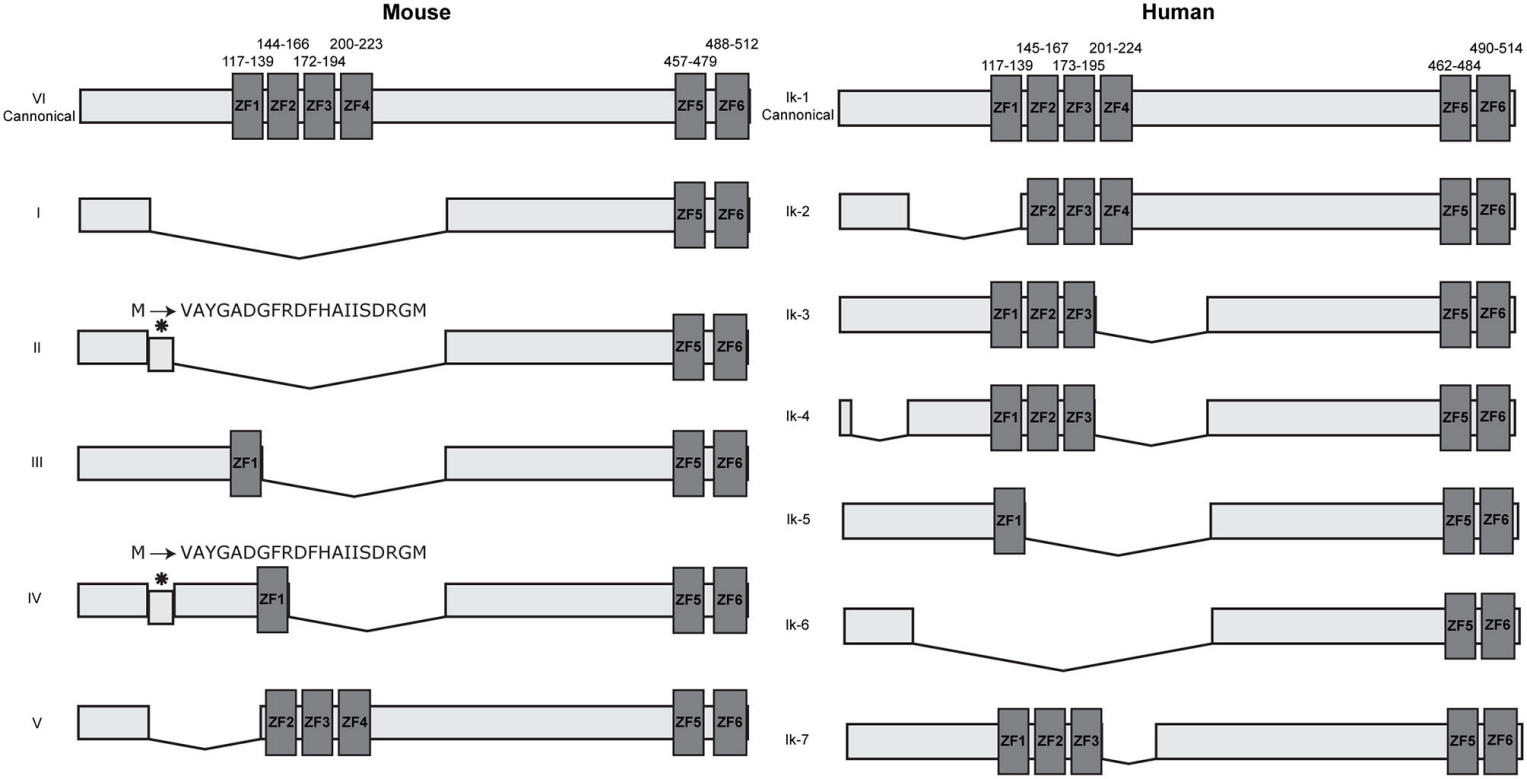
IKZF1 isoforms in mice and humans. IKZF1 contains six zinc finger domains, with the first four at the N terminus (ZF1–ZF4) responsible for DNA binding, and the last two at the C terminus (ZF5 & ZF6) responsible for protein dimerization. Dark gray boxes indicate zinc finger domains, small numbers above indicate amino acid locations for zinc fingers. Data obtained from UniProt (IKZF1-mouse: Q03267 and IKZF1-human: Q13422), isoform numbers may differ across other databases.

Multiple isoforms of IKZF1 have been described in both humans and mice, all products of alternative splicing. These isoforms differ in the number and combination of zinc finger domains and can be broadly split into two groups: DNA binding and non-DNA binding, based on whether the N-terminal zinc finger domains are present or not (2, 3, 30, 31). As the information available describing both murine and human IKZF1 isoforms are not free of discrepancies and scientific controversies, we will focus on the most generally accepted data to avoid overcomplicating this review. Dimers containing IKZF1 isoforms that lack a DNA-binding domain are transcriptionally inactive and are not able to activate gene transcription *in-vitro* (2). This generally suggests a dominant negative effect of the shorter isoforms that are non-DNA binding, yet later studies demonstrated that this is only observed when there is a drastic reduction in the amount of longer DNA binding isoforms available (7). Over six isoforms have been discovered in mice and humans with slight differences between murine and human isoforms (Figure 1). In mice, all isoforms maintain the C-terminal dimerization domain consisting of ZF5 and ZF6. Surprisingly, only two of the six murine isoforms contain a DNA binding domain with at least three N-terminal zinc fingers, compared with approximately half of the human isoforms. This would suggest that in mice, only the canonical isoform VI and potentially isoform V are functionally relevant as transcriptional activators as they are the only isoforms able to bind DNA with high affinity. A larger variety of isoforms have been discovered in humans, the majority of which are DNA binding isoforms that maintain at least three N-terminal zinc finger domains.

IKZF1 binds to pericentromeric repeats through direct DNA binding at pericentromeric-heterochromatin (PC-HC) (5) and is able to recruit repressed genes to PC-HC by binding directly to their promoter regions (7). It was originally hypothesized that PC-HC localization and promoter binding are exclusive events, as IKZF1 binding to pericentromeric repeats would obscure the DNA binding domain and prevent binding to target genes (5). Yet the ability of IKZF1 to both directly bind target genes and simultaneously bind to pericentromeric repeats was later shown to be facilitated through the formation of IKZF1 multimers consisting of individual IKZF1 monomers bound to either target genes or PC-HC (7). In endogenous systems, *IKZF1* can be alternatively spliced to produce multiple isoforms containing different combinations of each zinc finger (Figure 1) that are expressed during different stages of lymphoid development (3, 8, 30). These isoforms can interact with each other in different configurations, either as a homo or heterodimers, the outcome of which produces different functional activities (2, 31–34). Whilst the longest isoform mainly associates with PC-HC, some evidence suggests that other isoforms can direct transcriptional regulation outside of PC-HC (35). Furthermore, it was originally believed that shorter isoforms that do not contain the DNA binding domain disrupted the DNA binding ability of longer isoforms that do contain the N-terminal DNA binding domain (2, 5). Yet experiments by Trihn et al. (7) demonstrated that this in fact is not the case. Co-expressing murine isoforms VI and I in NIH3T3 cells and observing their nuclear location using immunofluorescence, showed localization of both isoforms at PC-HC. Yet over-expression of the shorter isoform I alone showed diffuse staining indicating an inability to localize to centromeric foci consistent with its lack of DNA binding domain (7), thus demonstrating that isoforms that lack the DNA binding domain are still able to associate with PC-HC when co-recruited by an isoform that contains a DNA binding domain. Furthermore, the expression of the smaller isoform I had no effect on DNA binding by endogenous IKZF1 isoforms (7). The authors therefore concluded that smaller isoforms that are unable to bind DNA are able to associate with larger isoforms in multimeric structures at PC-HC and that dominant negative effects of shorter isoforms are only apparent when the expression of the longer isoforms is lost.

## Murine Studies Into the Role of IKZF1

### Early Lymphocyte Development

Our understanding of IKZF1 biology has been greatly enhanced through the analysis of many *Ikzf1* mouse models [summarized in Heizmann et al. (36)]. The first reported *Ikzf1* mutant mice have a dominant negative mutation that removes ZF1, ZF2, and ZF3 in the DNA binding domain, rendering the protein unable to bind DNA (2, 25). Mice homozygous for this mutation (*Ikzf1*^*DN*/*DN*^) have a complete absence of all lymphoid progenitors in the fetal liver and postnatal bone marrow and have no mature B, T, or NK cells, thus defining IKZF1's role in the establishment of the lymphoid fate (25). Additionally, *Ikzf1*^*DN*/*DN*^ mice have only a very rudimentary thymus and no lymph nodes, and die at a very young age due to increased opportunistic infections and septicemia (25). Surprisingly, a few potential CD4 progenitors could be found in the rudimentary thymus in postnatal mice, potentially suggesting differential requirements of IKZF1 in T cell establishment compared with B cell establishment (25). Mice heterozygous for this mutation (*Ikzf1*^+/*DN*^) show no obvious B cell defects but spontaneously develop thymic derived leukemia as a result of the loss of the wildtype allele in clonally expanded populations (37). *Ikzf1*^*DN*/*DN*^ mice highlighted the crucial role IKZF1 plays in hematopoietic stem cell commitment to the lymphoid lineage, but *Ikzf1*^+/*DN*^ mice demonstrated the effects that mutant *Ikzf1* alleles can have on the functioning of wildtype Ikaros family members. By definition, the dominant negative nature of the *Ikzf1*^*DN*^ mutation meant that in a heterozygous state, the mutant IKZF1^DN^ protein could interfere with DNA binding of the wildtype IKZF1 protein, thus reducing the functionality of the wildtype protein (2, 37). This opened the door for exploring the effects that this type of mutation would have on the functioning of other Ikaros family members. It was plausible to assume that in a homozygous state, the IKZF1^DN^ protein could similarly be interfering with IKZF3 DNA binding (13), thus obscuring the sole effect of IKZF1 during lymphoid development.

In attempt to address this, the same group created an *Ikzf1*-null mouse (*Ikzf1*^−/−^) (26), where deleting exon 7 at the C-terminal dimerization domain resulted in the production of unstable null proteins that are not functional and are transcriptionally inactive (2). Wildtype IKZF1 isoforms VI and V (described as Ik-1 and Ik-2) were unable to be detected by western blot in thymocytes from *Ikzf1*^−/−^ mice (26). No lymphocyte progenitors could be found in the fetal liver (26) due to a reduction in HSC numbers and activity (38) leading to a subsequent absence of B, T, and NK cells. B cell precursors could not be found in the bone marrow of adult mice and subsequently, no mature B cells ever developed in *Ikzf1*^−/−^ mice (26). Conversely, low frequencies of T cell precursors were identified in the thymus of postnatal *Ikzf1*^−/−^ mice, despite there being no evidence of lymphocyte commitment in the fetal liver. Aberrant TCR signaling in the thymus (39) resulted in a skew toward CD4 single positive thymocytes at the expense of double positive thymocytes. As *Ikzf1*^−/−^ mice aged, T cells could be found at almost normal frequencies in the spleen, suggesting seeding from the thymus in adult mice was not affected (26). In support of this, γδ T cells that are normally found in the skin and epithelium that are seeded from the thymus during fetal development (40–45), were not present in *Ikzf1*^−/−^ mice, yet γδ T cells in the spleen and lymph nodes that are seeded from the thymus postnatally could be identified (26).

Unlike *Ikzf1*^*DN*/*DN*^ mice, *Ikzf1*^−/−^ mice are born at the expected frequency and survive into adulthood (26). Whilst *Ikzf1*^−/−^ mice were intended to overcome the issue of dominant negative effects of the *Ikzf1*^*DN*/*DN*^ mutation, the result was unfortunately not as clear-cut as first expected. It later became evident that removing IKZF1 expression entirely results in compensation from other Ikaros family members (31, 46, 47). Through both homodimerization and heterodimerization, IKZF1 creates macromolecular complexes that serve functionally different roles in the hematopoietic system (13, 14, 31, 48, 49). The complete removal of IKZF1 expression or function such as in *Ikzf1*^−/−^ and *Ikzf1*^*DN*/*DN*^ mice creates a hole in the repertoire that is potentially able to be filled by other Ikaros family members, thereby obscuring the direct role of IKZF1 in the hematopoietic system. This was demonstrated through the discovery of an ENU-induced point mutation in ZF3, H191R, that specifically disrupts DNA binding and localization to PC-HC but maintains protein scaffold structure and expression (46). When in homozygosity, the H191R mutation (*Ikzf1*^*Plstc*/*Plstc*^) creates a much more severe phenotype than the *Ikzf1*^−/−^ and *Ikzf1*^*DN*/*DN*^ mutations, with embryonic lethality at day E15.5 and a complete loss of B and T cell development in the fetal liver. Reconstitution experiments using either 100% *Ikzf1*^*Plstc*/*Plstc*^, or 50:50 *Ikzf1*^+/+^ and *Ikzf1*^*Plstc*/*Plstc*^ fetal liver cells failed to produce any macrophages, myeloid, or lymphoid cell subsets that were derived from the mutant cells. Heterozygous mice (*Ikzf1*^+/*Plstc*^) survive into adulthood but develop T cell lymphoma. A partial blockage of B cell development at the pro-B cell stage in the adult bone marrow also causes a reduction in mature B cell numbers in *Ikzf1*^+/*Plstc*^ mice. As all three mutations (*Ikzf1*^−/−^, *Ikzf1*^*DN*/*DN*^, and *Ikzf1*^*Plstc*/*Plstc*^) affected DNA binding and PC-HC localization, the difference in severity between the three mice models could not simply be accounted for by the absence of IKZF1 binding DNA. By preserving protein expression in *Ikzf1*^*Plstc*/*Plstc*^ mice, the niche that IKZF1 fills does not have to be maintained by other Ikaros family members and therefore the effects of the sole loss of IKZF1 functionality could account for the severity of the *Ikzf1*^*Plstc*/*Plstc*^ phenotype. Paradoxically, it was noted in Schjerven et al. (50) that when *Ikzf1*^−/−^ mice were attempted to be bred onto a pure C57BL/6 background, no homozygotes were born, likely due to embryonic lethality. This would suggest that the reported difference in severity between *Ikzf1*^−/−^ mice originally bred on a mixed 129SV background, and *Ikzf1*^*Plstc*/*Plstc*^ mice bred on a C57BL/6 background, could simply be due to genetic differences among background strains. Despite this, several other authors have reported differences in phenotypes between mice with the DN or null mutation when bred on the same mixed 129SV × C57BL/6 background (39, 51) suggesting differences in the underlying mechanism between the two mutations.

### Late Lymphocyte Development

It has become increasingly clear that IKZF1 is absolutely required for both T and B cell development, but exactly how IKZF1 discerns between these two transcriptional programs is still under debate. The absence of lymphopoiesis in the fetal liver of *Ikfz1*^*DN*/*DN*^, *Ikzf1*^−/−^, and *Ikzf1*^*Plstc*/*Plstc*^ mice, but presence of some lymphoid development in the thymus of surviving adult mice suggests differential requirements of IKZF1 in T and B cell commitment between the fetal liver and the bone marrow. Additionally, the thymus of *Ikzf1*^*Plstc*/*Plstc*^ embryos contains a decent proportion of B220^+^CD19^−^ precursor B cells in lieu of any Thy-1^+^ precursor T cells (46). This shift in T and B frequencies in the thymus affirmed previous suggestions that IKZF1 has a role in the regulation of T and B cell fate decisions.

The role of IKZF1 in B cell development was originally investigated by inserting a β-galactosidase reporter in-frame into the second exon of *Ikzf1*, which is present in all IKZF1 isoforms, just upstream of the N-terminal zinc finger domains (28). Mice homozygous for this mutation (*Ikzf1*^*L*/*L*^) lowly express a truncated form of IKZF1 and no full-length protein. Interestingly, this causes a complete block of B cell development in the fetal liver, and a partial blockage in the adult bone marrow between the pro- and pre-B cell stages. This was later shown to be because IKZF1 ensures B cell lineage commitment by positively regulating V_H_ rearrangements by directly binding to and activating *Rag1* and *Rag2* expression, as well as facilitating accessibility and compaction of the *Igh* locus (52). The few B cells that were able to successfully rearrange their BCR in *Ikzf1*^*L*/*L*^ mice surprisingly had a lower threshold for BCR activation *in-vitro*, and fewer germinal centers in response to bovine serum albumin (BSA) immunization, as well as reduced basal IgG3 secretion (28). These mice demonstrated that the low expression of a smaller IKZF1 protein can still establish B cell commitment in the postnatal bone marrow, compared with a complete loss of IKZF1 expression and/or function where B cell development is completely abolished.

To further this work, selectively removing either zinc finger 1 or zinc finger 4 demonstrated differential roles that each zinc finger plays in directing B and T cell development (50). Removing zinc finger 1 (*Ikzf1*^*ZF*1/*ZF*1^) induces a B cell specific defect with a partial block of development at the pro-B cell stage in the adult bone marrow, whilst deleting zinc finger 4 (*Ikzf1*^*ZF*4/*ZF*4^) prevents T cell development in the thymus and attenuation of development in large pre-B cells in the bone marrow. This would suggest that ZF4 is largely responsible for T cell development, but also has roles in B cell development, whilst ZF1 is exclusively responsible for B cell development. This work could also suggest that ZF1 and ZF4 are responsible for site-directed DNA binding but thus far, no DNA motifs that are specifically recognized by either ZF1 or ZF4 have been identified. Furthermore, the deletion of the entire zinc finger in these models has also removed the regulatory regions in the upstream flanking sequences (50), potentially impacting the interpretations of the independent roles each zinc finger has in regulating lineage-specific genes.

Recently, our group has described a mouse with a novel mutation in ZF1, L132P, that similarly to the *Plastic* mice have altered DNA binding capabilities but maintain full-length protein expression (53). Mice homozygous for this mutation, *Ikzf1*^*L*132*P*/*L*132*P*^, have reduced B cell development and an impaired T dependent humoral immune response, yet show no defects in T cell development. As the mutation lies within the first zinc finger, this provides further evidence that each zinc finger plays a different role in regulating lineage specific events.

With early lymphoid development in both the fetal liver and the adult bone marrow being dependent on IKZF1 expression, it has been impossible to determine the role of IKZF1 in mature B cell development and function. To achieve this, Schwickert et al. (29) created a conditional knock-out where *Ikzf1* is deleted in mature B cells only using *Cd23-Cre*. *Ikzf1*^*B*−^ mice have normal early B cell development, but develop splenomegaly and autoimmunity with elegant experiments demonstrating how IKZF1 maintains anergy induction in follicular B cells by directly regulating the anergic signature and also by restraining MyD88-dependent TLR signaling (29). Additionally, by conditionally knocking-out *Ikzf1* in the germinal center using *Aicda*-Cre, they demonstrated the crucial and intrinsic role of IKZF1 in the humoral immune response as *Ikzf1*^*GCB*−^, mice failed to elicit a strong germinal center and long-lived plasma cell response after T-dependent immunization (29).

Whilst collectively these mice demonstrate how even small amino acid substitutions to larger deletions of entire regions can cause widespread failure of the immune system, the differences between each mouse model highlight how complex IKZF1's involvement is from the earliest common lymphoid progenitor through until mature B cells. The mechanism of how IKZF1 aids different functions across the immune system by interacting not only with itself in different isoforms, but also with its family members, adds another layer of complexity that is yet to be fully explored. It is currently unknown how IKZF1 regulates such vastly different transcriptomes and how this shapes infection and immunity.

## IKZF1 in Human Disease

In recent years, our group and others have reported several different germline heterozygous mutations causing various immune defects in human patients [reviewed in (54–56)] leading to different forms of IKAROS-associated diseases. Since 2012, over 30 different variants in *IKZF1* have been identified across more than 100 individuals to cause various manifestations of immunological defects. The majority of these mutations lie within the functional regions of IKZF1, clustering mostly around ZF2 and ZF3 (part of the DNA binding domain) or ZF5 and ZF6 (the dimerization domain). Few mutations have been found outside of these functional domains and only one mutation has been reported in ZF1 (56, 57). Both large gene deletions and single missense mutations have been identified to cause immunological defects in individuals, the location of which can underly the pathomechanism of disease. Deletions that cause complete loss of protein expression leave only wildtype protein available to interact with itself thereby resulting in a haploinsufficient mechanism. Loss of function heterozygous missense variants also lead to haploinsufficiency. Depending on the location of the mutation, point mutations can affect either the DNA binding or dimerization properties of the IKZF1 protein. This can either result in the loss IKZF1 function of the mutant allele and retention of normal function of the wildtype allele and thus a haploinsufficient (HI) mode of disease, or it can result in the mutant protein negatively impacting the function of the wildtype protein and the complete loss of IKZF1 function, resulting in a dominant negative (DN) mode of disease. In some cases, the mutant IKZF1 can negatively affect the functioning of other Ikaros family members, such as IKZF3, in a dominant negative fashion (58). Lastly, mutations located in or directly affecting the dimerization domain that negatively impact dimerization in a haploinsufficient manner are classified as dimerization defective (DD) mutations (59).

DN mutations generally present with more severe disease manifestations compared with HI mutations (54, 55), presenting as severe and early onset combined immunodeficiency in eight patients, and a milder phenotype with antibody deficiency in one patient (60). Of the nine individuals identified, eight of those carry the same N159S mutation with the ninth patient carrying a different substitution affecting the same amino acid, N159T. The N159S/T mutations prevent both the mutant and wildtype protein localizing to PC-HC, yet maintain normal homodimerization and heterodimerization with IKZF3 (58). Additionally, the mutant protein also interferes with the localization of IKZF3 to PC-HC (58). Functionally, the N159S/T mutations cause severe defects in early B cell development resulting in an almost complete absence of B cells in the periphery and absent plasma cells with all patients developing profound hypogammaglobulinemia. T cell defects arising through skewed frequencies of naïve and memory/effector populations as well as impaired responses to TCR stimulation *in-vitro* are also present in individuals with *IKZF1* DN mutations. Recurrent and severe infections to *Pneumocystis jirovecii* and a broad range of bacterial, viral, and other fungal pathogens have been reported in most DN patients. While hematologic malignancies (e.g., T-cell acute lymphoblastic leukemia) were reported among these patients, immune dysregulation was not. Hematopoietic stem cell transplantation were curative for this severe form of IKAROS-associated disease (61).

HI mutations are more frequently reported but cause a less severe phenotype than DN mutations. To date, more than 16 different HI mutations have been reported in patients with common variable immunodeficiency (CVID) phenotypes. More than 65 individuals have shown to carry the HI mutations and about one third of them are asymptomatic mutation carriers (54, 56). The presence of asymptomatic cases in HI *IKZF1* mutations compared with DN mutations, suggests either incomplete penetrance or delayed onset of disease. Bacterial infections, mainly *Streptococcus pneumoniae*, are most commonly reported in CVID patients with *IKZF1* HI mutations, mainly affecting the respiratory tract. Reduced B cells and a progressive decline of serum IgG are present in the majority of HI patients, with T cell counts in the normal to high range. Immune dysregulation (e.g., immune thrombocytopenia) and hematologic malignancies (e.g., B-cell acute lymphoblastic leukemia) were also reported in this form of the disease (Table 1).

**Table 1:** Key findings of heterozygous *IKZF1* germline mutations in human disease and corresponding mouse models.

	**Allelic variants**			
	**Haploinsufficiency through DNA binding defect (WT/null or WT/mis expression)**	**Haploinsufficiency through Dimerization defect**	**Dominant Negative (N159S/T) through DNA binding defect**	**Mouse models**
**Clinical phenotype**				
Infections	Recurrent, variable severity Sinopulmonary are the most frequent; *Streptococcus pneumoniae* most commonly isolated Mostly bacterial, small subset of patients with mild viral infections	Recurrent in ~20% of patients, neither severe nor invasive Sinopulmonary are the most frequent Bacterial	Early onset, recurrent and severe Sinopulmonary, skin abscesses, meningitis; *Pneumocystis jirovecii* pneumonia; Bacterial, viral (RSV, HSV, adenovirus, influenza, molluscum), mycobacterial, parasitic	Septicemia leading to early mortality in *Ikzf1^*DN*/*DN*^* mice
Autoimmunity/ immune dysregulation	Present in ~1/3 of patients Commonly of childhood onset ITP, SLE, arthritis, APLS	Present in ~1/2 of patients Presentation usually in the first decade of life ITP, AIHA, neutropenia, Evans syndrome	Not reported	*Ikzf1^*B*−^* mice develop systemic autoimmunity due to lack of B cell tolerance
Malignancy	Incidence: 6.2% B-ALL (3 patients) Solid pancreatic tumor (1 patient)	Incidence: 20% T-ALL (1 patient) B-ALL (1 patient) Burkitt Lymphoma (1 patient)	Incidence: 11.1% T-ALL (1 patient)	*Ikzf1^−^*^/−^ have high penetrance of T-cell leukemia/lymphoma Heterozygous *Ikzf1^+/*DN*^* T cell malignancies a few months after birth Heterozygous *Ikzf1^+/*Plstc*^* T cell leukemia a few months after birth
**Immunological phenotype**				
**Lymphoid lineage**				
B cells	Low B cells (70%) Low IgG, IgA, IgM Progressive decline of B cell numbers and immunoglobulin levels	Low B cells (~36%) Low IgG, IgA, IgM	B cells nearly absent Profoundly low IgG, IgA, IgM	Absence of B cells in the fetal liver or adult bone marrow; no mature B cells in *Ikzf1^−/−^* Low B cell numbers and failure to mount proper humoral immune response in *Ikzf1^*L*132*P*/*L*132*P*^* and *Ikzf1^+/*L*132*P*^* Reduced in *Ikzf1^*ZF*1/*ZF*1^* and *Ikzf1^*ZF*4/*ZF*4^* Complete block of fetal B cell development and partial block in post-natal bone marrow in *Ikzf1^*L*/*L*^* Absent in *Ikzf1^*Plstc*/*Plstc*^*, partially blocked at pro-B cell stage in post-natal bone marrow of *Ikzf1^+/*Plstc*^* mice Absent in *Ikzf1^*DN*/*DN*^* Normal B cell numbers in *Ikzf1^+/*DN*^* 1 month after birth
T cells	Normal or high T cell numbers in the periphery Trend toward increased CD8+ with inverted CD4/CD8 ratio (mostly in missense mutations)	Normal or high T cell numbers	Variable counts Predominance of phenotypically naïve cells Reduction in memory/effector T cells Failure to acquire a memory phenotype after *in vitro* stimulation Impaired T cell proliferation in response to weak TCR stimuli	Absence of fetal T cells in *Ikzf1^−/−^*, aberrant post-natal development skewed toward CD4^+^; clonal expansion results in normal T cell numbers in adult spleen Absent in *Ikzf1^*Plstc*/*Plstc*^* and partially blocked in *Ikzf1^+/*Plstc*^* thymi Reduced in *Ikzf1^*ZF*4/*ZF*4^* Absent in *Ikzf1^*DN*/*DN*^* Normal T cell numbers in *Ikzf1^+/*DN*^* 1 month after birth
NK cells	Reduced in ~1/5 of patients (mostly in missense mutations)	Normal or low	Normal or low	Absence of fetal NK cells in *Ikzf1^−/−^* Absent in *Ikzf1^*DN*/*DN*^* Normal NK cell numbers in *Ikzf1^+/*DN*^* 1 month after birth NK cell deficiency is one of the main features of Ikaros transgenic mice
**Myeloid lineage**				
	Skewed DC subset differentiation: reduction in pDC and increase in cDC1		Eosinopenia; neutropenia Monocyte dysfunction in *in vitro* studies Mild reduction in pDC	*Ikzf1^−/−^* mice – severe reduction in thymic dendritic cells Specific defect in pDC in *Ikzf1^*L*/*L*^* Terminal granulocyte differentiation defective in *Ikzf1^*Plstc*/*Plstc*^* *Ikzf1^*DN*/*DN*^* normal production of myeloid lineages but a complete lack of DC in lymphoid organs (thymus and spleen)

*RSV, respiratory syncytial virus; HSV, herpes simplex virus; DN, dominant negative; ITP, idiopathic thrombocytopenic purpura; SLE, systemic lupus erythematosus; APLS, antiphospholipid antibody syndrome; AIHA, autoimmune hemolytic anemia; B-ALL, B cell acute lymphoblastic leukemia; T-ALL, T cell acute lymphoblastic leukemia; TCR, T cell receptor; NK, natural killer cell; DC, dendritic cell; pDC, plasmacytoid dendritic cell; cDC1, conventional dendritic cell 1*.

Recently, in a multicenter international collaboration, a novel allelic variant associated with *IKZF1* DD mutations causing mostly hematological disorders with limited infectious disease susceptibility in four unrelated families was reported by our group (59). These DD variants are located towards or compromise the C-terminus of the protein and affect homo- and hetero- dimerization of IKZF1. Clinically, patients presented with a range of symptoms including hematopoietic cytopenias (some presented with Evans syndrome), various hematologic malignancies, immune dysregulation and hypogammaglobulinemia. Few patients suffered from increased susceptibility to infections (Table 1). Surprisingly, one of the four mutations identified (R502L) located in ZF6, was still able to form dimers at a significantly reduced rate with wildtype IKZF1 and had only moderately decreased heterodimerization with wildtype IKZF2 and IKZF3. This resulted in the ability of the mutant protein to bind PC-HC, whilst the other 3 DD mutations had lost this ability. Direct DNA-binding was still achievable in all DD mutants, yet only the R502L mutation allowed for DNA binding as a monomer and dimer, the others were only able to bind as monomers. Additionally, partial dimerization of the R502L mutant allowed for normal repression of gene transcription, whilst the other DD mutations could not do so *in-vitro*. Protein stability was affected in all DD but not other allelic variants such as HI and DN mutations.

## The Complexity of Understanding IKZF1 in Humans and Mice

The wide variety of pathogenicity of IKZF1-related diseases suggests different underlying mechanisms that are related to the location and effect of the mutation on protein function (54, 55). The molecular effect on DNA binding to chromatin and the formation of dimeric complexes has been characterized in most known mutations (55). These analyses have shed light on how different single point mutations can have vastly different outcomes in terms of protein function. Analyses in mice and humans clearly show that IKZF1 works in a complex and intricate system by not only forming complexes with itself, but also with four other family members (13). Another added layer of complexity lies within the different modes of action that control either activation or repression of gene transcription. IKZF1 can regulate genes at the local level by binding with the promoter regions of target genes (2–4). More globally, it can regulate the chromatin landscape of the cell through acting as a transcription factor to open up chromatin, forming poised or active enhances, or by deleting enhancers to repress gene transcription (4–11, 62). The downstream effects of the multitude of *IKZF1* point mutations on site-directed gene transcription is yet to be fully elucidated. How these mutations affect the ability to bind to specific DNA sequences is unknown and would require large-scale molecular modeling combined with RNA and CHIPseq in both B and T cells. The broad range of clinical defects observed in patients with *IKZF1* mutations suggests that specific point mutations can have selective effects on some parts of the gene expression profile. Furthermore, the effects of the loss of heterodimeric complexes as opposed to homodimeric complexes has not yet been determined. Isolated loss-of-function experiments in mice have indicated vastly different roles for Ikaros family members in leukocyte development and function (63) yet have also highlighted some redundancy and compensatory mechanisms (15, 46, 64) that obscure the role of each individual family member.

Additionally, there is sufficient evidence in mice to suggest that each zinc finger has a differential role in directing lineage specific functions. ZF1 and ZF4 allow for differential targeting of DNA sequences in mice *in-vivo*; ZF1 appears to bind specifically to repeat motifs flanking the Ikaros consensus sequence whilst ZF4 appears to be important for stabilizing DNA-interactions made by ZF2 and ZF3 (50). Consequently, mutations in ZF1 alone cause B cell defects (53), yet mutations in ZF4 cause defects in both B and T cells (50). This phenomenon however, has not yet been explored in humans as thus far only one mutation in ZF1 has been identified (D120V) in an individual presenting with systemic lupus erythematosus (SLE), but as they also carry a likely deleterious heterozygous mutation in *LYN*, it is difficult to isolate the *IKZF1* mutation as the sole cause of disease (57). Whether the absence of striking ZF1 mutations in humans speaks to the differences between mouse and human IKZF1, Ikaros family members as a whole, or even more broad-scale differences between mice and humans in general have yet to be determined. Mutations in ZF4 in humans are less common than those in ZF2 or ZF3 and there is limited data to suggest that ZF1 and ZF4 are able to control site-directed DNA binding as is the case in mice (50). Another layer of complexity exists when one considers the effects of single point mutations in each zinc finger, as is observed in human patients, vs. the effect of completely removing an entire zinc finger including the surrounding flanking sequences as studies performed in mice have done (50). There is already mounting evidence that individual point mutations can cause very different functional and pathological outcomes in humans (54) and this would suggest that even small changes to how IKZF1 interacts with DNA can have different downstream effects. Collectively comparing heterozygous point mutations in humans to null mutations in mice is therefore not an accurate comparison.

Lastly, the biological relevance of multiple isoforms existing in multimeric structures must not be ignored when considering the molecular impact of *IKZF1* mutations. Thus far, all molecular studies into human *IKZF1* mutations and their effects on protein-protein interactions and DNA binding at PC-HC *in-vitro* have been performed on the human Ik-1 isoform. In the case of heterozygous HI mutations, the presence of one wildtype IKZF1 is sufficient to allow for PC-HC association and DNA binding in exclusive *in-vitro* systems. In these scenarios, the mutant protein is still recruited to pericentromeric foci as a multimer with wildtype Ik-1. DN mutations however, abolish the ability of the wildtype Ik-1 protein to localize to PC-HC (58). Yet in patients with mutations in *IKZF1* as well as in mice with *Ikzf1* mutations, there will be endogenous expression of isoforms that do not contain the mutant allele *in-vivo*. The multimerization of mutant isoforms with these otherwise “wildtype” isoforms *in-vivo* has not yet been explored. Given that the lack of the DNA-binding domain does not necessarily negate protein function in certain isoforms (7), the absence of these isoforms in the exclusive *in-vitro* systems in which these mutations were analyzed could be falsely producing binary on and off answers in an otherwise complex setting. Whilst the two longest isoforms (VI and V in mice, and Ik-1 and Ik-2 in humans) are the most highly expressed within the lymphoid lineages, the concentration of other isoforms relative to the longer ones changes throughout lymphoid development (1, 3, 15). The configuration in which they form multimers and interact with each other can produce different functional consequences on gene expression (3, 30, 35). Additionally, the way in which different IKZF1 isoforms interact with isoforms from other family members such as IKZF2 and IKZF3, further increases the diversity of multimer formation influencing downstream DNA binding effects (31–34). Due to the compensatory nature of Ikaros family members and their different prevalence across multiple cell types (31, 33), over-expressing only one isoform of a mutant version of IKZF1 does not necessarily give the whole picture as to what is happening *in-vivo* and how other family members could be compensating for certain defects across different cell types. It is therefore possible that multimers consisting of different configurations of mutant and/or wildtype isoforms have the ability to change the overall IKZF1-regulated landscape *in-vivo*.

### From Humans to Mice

IKZF1 acts in a complex and dynamic network *in-vivo*, yet current attempts to understand the molecular mechanism of IKZF1 mutations are occurring in isolated *in-vitro* systems. The exclusive analysis of overexpression systems, whereby, specific point mutations that have been discovered in human patients are artificially expressed *in-vitro*, does not necessarily or completely pinpoint the underlying molecular defect causing the full spectrum of the clinical phenotype of the patient. To understand these biological networks in the context of mammalian disease, *in-vivo* studies demonstrating cause and effect of known *IKZF1* mutations in *in-vivo* systems could be required. The majority of existing murine models of *Ikzf1* mutations take a sledge-hammer approach, whereby, removal of the entire gene or segments of the gene, causes significant disruptions to the expression of the protein. This effect of the loss of protein structure and/or expression is however, not reflected in the majority of human *IKZF1* mutations (54, 55). In general, the loss or reduction of IKZF1 expression in mice causes severe phenotypes. *Ikzf1*^−/−^ mice have no B cells and very few T cells (26), whilst *Ikzf1*^*DN*/*DN*^ mice have a complete absence of lymphocytes and die prematurely (2). Selectively removing sections of *Ikzf1* to either reduce its expression (*Ikzf1*^*L*/*L*^) (27), or reduce the specific activity of IKZF1 (*Ikzf1*^*ZF*1/*ZF*1^ and *Ikzf1*^*ZF*4/*ZF*4^) (50) also have broader and more severe defects than those observed in human patients with heterozygous *IKZF1* mutations. Conversely, a single point mutation in ZF3 that still maintains correct protein expression leads to a severe phenotype with embryonic lethality when in homozygosity as well as complete absence of lymphoid cell development (46). Phenotypes to this extreme are not commonly observed in human patients with *IKZF1* mutations, and whilst there are some phenotypes that are reproduced in both humans and mice expressing mutant IKZF1 alleles, the majority of more subtle clinical features arising from human *IKZF1* mutations have not been replicated in murine models (Table 1). An exception to this however, is the *Ikzf1*^+/*L*132*P*^ mouse, where a heterozygous missense mutation in ZF1 leads to a decrease in mature B cells and progressive decline in immunoglobulin titers as well as a failure to elicit a robust humoral immune response (53). These features are commonly observed in patients with *IKZF1* mutations and demonstrates how specific point mutations, rather than entire gene deletions, are more equipped to study the molecular effects of IKZF1 mutant alleles.

Through the use of CRISPR-Cas9, the study of specific point mutations originally discovered in human patients that have been redeveloped into the murine germline has been increasing in popularity (65, 66). This allows for the exact replication of specific point mutations in mice and facilitates the study of how these mutant alleles function in an *in-vivo* system, that has as of yet been unexplored in the context of *IKZF1-*related diseases. Additionally, maintaining the endogenous repertoire of Ikaros family members preserves these interactions in a biologically relevant way. Analysis of the changes these mutations make to the RNA and chromatin landscape, combined with analysis of site-directed DNA binding outside of heterochromatin and downstream phenotypic effects will allow for an in-depth characterization of the effect of individual point mutations in *IKZF1* causing immune defects in human patients.

## Conclusion

IKZF1 is a complex protein in how it interacts not only with DNA, but also with itself and with its family members to create a large pool of dimers and multimers that have the potential to change the RNA and chromatin landscape of a cell. The ability for some family members to compensate for the loss of others further obscures the function of each individual family member. Additionally, the dependence on IKZF1 during very different stages of lymphoid, B, T, and myeloid cell development further complicates how we assess the functional relevance of this protein when in the context of human disease. The diverse phenotypes observed as a consequence of *IKZF1* mutations in humans only attests to this deeply complex family of immune regulators. To understand how these human variants are affecting gene transcription and leading to functionally different outcomes, mouse studies linking human mutations and phenotypes are required. The limitations with artificial *in-vitro* systems therefore call for a systematic investigation of *in-vivo* mechanisms of IKZF1 both in the context of wildtype and mutant alleles.

## Author Contributions

BB wrote the first draft. CJN-S, HSK, and SDR contributed to the work. SDR supervised the project. All authors contributed to the article and approved the submitted version.

## Conflict of Interest

The authors declare that the research was conducted in the absence of any commercial or financial relationships that could be construed as a potential conflict of interest.

## References

[1] Molnár Á Wu P Largespada DA Vortkamp A Scherer S Copeland NG. The Ikaros gene encodes a family of lymphocyte-restricted zinc finger DNA binding proteins, highly conserved in human and mouse. J Immunol. (1996) 156:585–92. 8543809

[2] Sun L Liu A Georgopoulos K. Zinc finger-mediated protein interactions modulate Ikaros activity, a molecular control of lymphocyte development. EMBO J. (1996) 15:5358. 10.1002/j.1460-2075.1996.tb00920.x 8895580 PMC452279

[3] Molnar A Georgopoulos K. The Ikaros gene encodes a family of functionally diverse zinc finger DNA-binding proteins. Mol Cell Biol. (1994) 14:8292–303. 10.1128/mcb.14.12.8292-8303.1994 7969165 PMC359368

[4] Schwickert TA Tagoh H Gültekin S Dakic A Axelsson E Minnich M. Stage-specific control of early B cell development by the transcription factor Ikaros. Nat Immunol. (2014) 15:283. 10.1038/ni.2828 24509509 PMC5790181

[5] Cobb BS Morales-Alcelay S Kleiger G Brown KE Fisher AG Smale ST. Targeting of Ikaros to pericentromeric heterochromatin by direct DNA binding. Genes Dev. (2000) 14:2146–60. 10.1101/gad.816400 10970879 PMC316893

[6] Kim J Sif S Jones B Jackson A Koipally J Heller E. Ikaros DNA-binding proteins direct formation of chromatin remodeling complexes in lymphocytes. Immunity. (1999) 10:345–55. 10.1016/S1074-7613(00)80034-5 10204490

[7] Trinh LA Ferrini R Cobb BS Weinmann AS Hahm K Ernst P. Down-regulation of TDT transcription in CD4+ CD8+ thymocytes by Ikaros proteins in direct competition with an Ets activator. Genes Dev. (2001) 15:1817–32. 10.1101/gad.905601 11459831 PMC312741

[8] Brown KE Guest SS Smale ST Hahm K Merkenschlager M Fisher AG. Association of transcriptionally silent genes with Ikaros complexes at centromeric heterochromatin. Cell. (1997) 91:845–54. 10.1016/S0092-8674(00)80472-9 9413993

[9] Koipally J Renold A Kim J Georgopoulos K. Repression by Ikaros and Aiolos is mediated through histone deacetylase complexes. EMBO J. (1999) 18:3090–100. 10.1093/emboj/18.11.3090 10357820 PMC1171390

[10] Sridharan R Smale ST. Predominant interaction of both Ikaros and Helios with the NuRD complex in immature thymocytes. J Biol Chem. (2007) 282:30227–38. 10.1074/jbc.M702541200 17681952

[11] Koipally J Georgopoulos K. A molecular dissection of the repression circuitry of Ikaros. J Biol Chem. (2002) 277:27697–705. 10.1074/jbc.M201694200 12015313

[12] Georgopoulos K Moore DD Derfler B. Ikaros, an early lymphoid-specific transcription factor and a putative mediator for T cell commitment. Science. (1992) 258:808–12. 10.1126/science.1439790 1439790

[13] Morgan B Sun L Avitahl N Andrikopoulos K Ikeda T Gonzales E. Aiolos, a lymphoid restricted transcription factor that interacts with Ikaros to regulate lymphocyte differentiation. EMBO J. (1997) 16:2004–13. 10.1093/emboj/16.8.2004 9155026 PMC1169803

[14] Kelley CM Ikeda T Koipally J Avitahl N Wu L Georgopoulos K. Helios, a novel dimerization partner of Ikaros expressed in the earliest hematopoietic progenitors. Curr Biol. (1998) 8:508–15. 10.1016/S0960-9822(98)70202-7 9560339

[15] Klug CA Morrison SJ Masek M Hahm K Smale ST Weissman IL. Hematopoietic stem cells and lymphoid progenitors express different Ikaros isoforms, and Ikaros is localized to heterochromatin in immature lymphocytes. Proc Nat Acad Sci. (1998) 95:657–62. 10.1073/pnas.95.2.657 9435248 PMC18476

[16] Adolfsson J Månsson R Buza-Vidas N Hultquist A Liuba K Jensen CT. Identification of Flt3+ lympho-myeloid stem cells lacking erythro-megakaryocytic potential: a revised road map for adult blood lineage commitment. Cell. (2005) 121:295–306. 10.1016/j.cell.2005.02.013 15851035

[17] Adolfsson J Borge OJ Bryder D Theilgaard-Mönch K Åstrand-Grundström I Sitnicka E. Upregulation of Flt3 expression within the bone marrow Lin– Sca1+ c-kit+ stem cell compartment is accompanied by loss of self-renewal capacity. Immunity. (2001) 15:659–69. 10.1016/S1074-7613(01)00220-5 11672547

[18] Kondo M Weissman IL Akashi K. Identification of clonogenic common lymphoid progenitors in mouse bone marrow. Cell. (1997) 91:661–72. 10.1016/S0092-8674(00)80453-5 9393859

[19] von Freeden-Jeffry U Vieira P Lucian LA McNeil T Burdach S Murray R. Lymphopenia in interleukin (IL)-7 gene-deleted mice identifies IL-7 as a non-redundant cytokine. J Exp Med. (1995) 181:1519–26. 10.1084/jem.181.4.1519 7699333 PMC2191954

[20] Miller JP Izon D DeMuth W Gerstein R Bhandoola A Allman D. The earliest step in B lineage differentiation from common lymphoid progenitors is critically dependent upon interleukin 7. J Exp Med. (2002) 196:705–11. 10.1084/jem.20020784 12208884 PMC2193997

[21] Peschon JJ Morrissey PJ Grabstein KH Ramsdell FJ Maraskovsky E Gliniak BC. Early lymphocyte expansion is severely impaired in interleukin 7 receptor-deficient mice. J Exp Med. (1994) 180:1955–60. 10.1084/jem.180.5.1955 7964471 PMC2191751

[22] Prieyl J LeBien TW. Interleukin 7 independent development of human B cells. Proc Nat Acad Sci. (1996) 93:10348–53. 10.1073/pnas.93.19.10348 8816803 PMC38387

[23] Kondo M Scherer DC Miyamoto T King AG Akashi K Sugamura K. Cell-fate conversion of lymphoid-committed progenitors by instructive actions of cytokines. Nature. (2000) 407:383. 10.1038/35030112 11014194

[24] Busslinger M. Transcriptional control of early B cell development. Annu Rev Immunol. (2004) 22:55–79. 10.1146/annurev.immunol.22.012703.104807 15032574

[25] Georgopoulos K Bigby M Wang J-H Molnar A Wu P Winandy S. The Ikaros gene is required for the development of all lymphoid lineages. Cell. (1994) 79:143–56. 10.1016/0092-8674(94)90407-3 7923373

[26] Wang J-H Nichogiannopoulou A Wu L Sun L Sharpe AH Bigby M. Selective defects in the development of the fetal and adult lymphoid system in mice with an Ikaros null mutation. Immunity. (1996) 5:537–50. 10.1016/S1074-7613(00)80269-1 8986714

[27] Yoshida T Ng SY-M Zuniga-Pflucker JC Georgopoulos K. Early hemopoietic lineage restrictions directed by Ikaros. Nat Immunol. (2006) 7:382–91. 10.1038/ni1314 16518393 PMC3872276

[28] Kirstetter P Thomas M Dierich A Kastner P Chan S. Ikaros is critical for B cell differentiation and function. Eur J Immunol. (2002) 32:720–30. 10.1002/1521-4141(200203)32:3<720::AID-IMMU720>3.0.CO;2-P 11870616

[29] Schwickert TA Tagoh H Schindler K Fischer M Jaritz M Busslinger M. Ikaros prevents autoimmunity by controlling anergy and Toll-like receptor signaling in B cells. Nat Immunol. (2019) 20:1517–29. 10.1038/s41590-019-0490-2 31591571 PMC7115902

[30] Hahm K Ernst P Lo K Kim GS Turck C Smale ST. The lymphoid transcription factor LyF-1 is encoded by specific, alternatively spliced mRNAs derived from the Ikaros gene. Mol Cell Biol. (1994) 14:7111–23. 10.1128/mcb.14.11.7111-7123.1994 7935426 PMC359245

[31] Hahm K Cobb BS McCarty AS Brown KE Klug CA Lee R. Helios, a T cell-restricted Ikaros family member that quantitatively associates with Ikaros at centromeric heterochromatin. Genes Dev. (1998) 12:782–96. 10.1101/gad.12.6.782 9512513 PMC316626

[32] Dovat S Montecino-Rodriguez E Schuman V Teitell MA Dorshkind K Smale ST. Transgenic expression of Helios in B lineage cells alters B cell properties and promotes lymphomagenesis. J Immunol. (2005) 175:3508–15. 10.4049/jimmunol.175.6.3508 16148093

[33] Alinikula J Kohonen P Nera KP Lassila O. Concerted action of Helios and Ikaros controls the expression of the inositol 5-phosphatase SHIP. Eur J Immunol. (2010) 40:2599–607. 10.1002/eji.200940002 20602434

[34] Li Z Perez-Casellas LA Savic A Song C Dovat S. Ikaros isoforms: the saga continues. World J Biol Chem. (2011) 2:140. 10.4331/wjbc.v2.i6.140 21765980 PMC3135861

[35] Ronni T Payne KJ Ho S Bradley MN Dorsam G Dovat S. Human Ikaros function in activated T cells is regulated by coordinated expression of its largest isoforms. J Biol Chem. (2007) 282:2538–47. 10.1074/jbc.M605627200 17135265

[36] Heizmann B Kastner P Chan S. The Ikaros family in lymphocyte development. Curr Opin Immunol. (2018) 51:14–23. 10.1016/j.coi.2017.11.005 29278858

[37] Winandy S Wu P Georgopoulos K. A dominant mutation in the Ikaros gene leads to rapid development of leukemia and lymphoma. Cell. (1995) 83:289–99. 10.1016/0092-8674(95)90170-1 7585946

[38] Nichogiannopoulou A Trevisan M Neben S Friedrich C Georgopoulos K. Defects in hemopoietic stem cell activity in Ikaros mutant mice. J Exp Med. (1999) 190:1201–14. 10.1084/jem.190.9.1201 10544193 PMC2195677

[39] Winandy S Wu L Wang J-H Georgopoulos K. Pre–T cell receptor (TCR) and TCR-controlled checkpoints in T cell differentiation are set by Ikaros. J Exp Med. (1999) 190:1039–48. 10.1084/jem.190.8.1039 10523602 PMC2195663

[40] Havran WL Allison JP. Origin of Thy-1+ dendritic epidermal cells of adult mice from fetal thymic precursors. Nature. (1990) 344:68–70. 10.1038/344068a0 1968230

[41] Koning F Stingl G Yokoyama WM Yamada H Maloy WL Tschachler E. Identification of a T3-associated gamma delta T cell receptor on Thy-1+ dendritic epidermal Cell lines. Science. (1987) 236:834–7. 10.1126/science.2883729 2883729

[42] Asarnow DM Kuziel WA Bonyhad M Tigelaar RE Tucker PW Allison JP. Limited diversity of γδ antigen receptor genes of Thy-1+ dendritic epidermal cells. Cell. (1988) 55:837–47. 10.1016/0092-8674(88)90139-0 2847872

[43] Havran WL Allison JP. Developmentally ordered appearance of thymocytes expressing different T-cell antigen receptors. Nature. (1988) 335:443–5. 10.1038/335443a0 2458531

[44] Ito K Bonneville M Takagaki Y Nakanishi N Kanagawa O Krecko EG. Different gamma delta T-cell receptors are expressed on thymocytes at different stages of development. Proc Nat Acad Sci. (1989) 86:631–5. 10.1073/pnas.86.2.631 2463632 PMC286526

[45] Itohara S Farr AG Lafaille JJ Bonneville M Takagaki Y Haas W. Homing of a γδ thymocyte subset with homogeneous T-cell receptors to mucosal epithelia. Nature. (1990) 343:754–7. 10.1038/343754a0 2154700

[46] Papathanasiou P Perkins AC Cobb BS Ferrini R Sridharan R Hoyne GF. Widespread failure of hematolymphoid differentiation caused by a recessive niche-filling allele of the Ikaros transcription factor. Immunity. (2003) 19:131–44. 10.1016/S1074-7613(03)00168-7 12871645

[47] Schmitt C Tonnelle C Dalloul A Chabannon C Debre P Rebollo A. Aiolos and Ikaros: regulators of lymphocyte development, homeostasis and lymphoproliferation. Apoptosis. (2002) 7:277–84. 10.1023/A:1015372322419 11997672

[48] Honma Y Kiyosawa H Mori T Oguri A Nikaido T Kanazawa K-y. Eos: a novel member of the Ikaros gene family expressed predominantly in the developing nervous system. FEBS Lett. (1999) 447:76–80. 10.1016/S0014-5793(99)00265-3 10218586

[49] Perdomo J Holmes M Chong B Crossley M. Eos and pegasus, two members of the Ikaros family of proteins with distinct DNA binding activities. J Biol Chem. (2000) 275:38347–54. 10.1074/jbc.M005457200 10978333

[50] Schjerven H McLaughlin J Arenzana TL Frietze S Cheng D Wadsworth SE. Selective regulation of lymphopoiesis and leukemogenesis by individual zinc fingers of Ikaros. Nat Immunol. (2013) 14:1073–83. 10.1038/ni.2707 24013668 PMC3800053

[51] Avitahl N Winandy S Friedrich C Jones B Ge Y Georgopoulos K. Ikaros sets thresholds for T cell activation and regulates chromosome propagation. Immunity. (1999) 10:333–43. 10.1016/S1074-7613(00)80033-3 10204489

[52] Reynaud D Demarco IA Reddy KL Schjerven H Bertolino E Chen Z. Regulation of B cell fate commitment and immunoglobulin heavy-chain gene rearrangements by Ikaros. Nat Immunol. (2008) 9:927–36. 10.1038/ni.1626 18568028 PMC2699484

[53] Boast B Miosge LA Kuehn HS Cho V Athanasopoulos V McNamara HA. A point mutation in IKAROS ZF1 causes a B cell deficiency in mice. J Immunol. (2021) 206:1505–14. 10.4049/jimmunol.1901464 33658297 PMC7987828

[54] Nunes-Santos CJ Kuehn HS Rosenzweig SD. IKAROS family zinc finger 1–associated diseases in primary immunodeficiency patients. Immunol Allergy Clin. (2020) 40:461–70. 10.1016/j.iac.2020.04.004 32654692 PMC7394939

[55] Kuehn HS Nunes-Santos CJ Rosenzweig SD. IKAROS-associated diseases in 2020: genotypes, phenotypes, and outcomes in primary immune deficiency/inborn errors of immunity. J Clin Immunol. (2021) 41:1–10. 10.1007/s10875-020-00936-x 33392855

[56] Kuehn HS Nunes-Santos CJ Rosenzweig SD. Germline IKZF1mutations and their impact on immunity: IKAROS-associated diseases and pathophysiology. Expert Rev Clin Immunol. (2021) 17:407–16. 10.1080/1744666X.2021.1901582 33691560 PMC8091572

[57] Belot A Rice GI Omarjee SO Rouchon Q Smith EM Moreews M. Contribution of rare and predicted pathogenic gene variants to childhood-onset lupus: a large, genetic panel analysis of British and French cohorts. Lancet Rheumatol. (2020) 2:e99–109. 10.1016/S2665-9913(19)30142-0 38263665

[58] Boutboul D Kuehn HS Van de Wyngaert Z Niemela JE Callebaut I Stoddard J. Dominant-negative IKZF1 mutations cause a T, B, and myeloid cell combined immunodeficiency. J Clin Invest. (2018) 128:3071–87. 10.1172/JCI98164 29889099 PMC6026000

[59] Kuehn HS Niemela JE Stoddard J Mannurita SC Shahin T Goel S. Germline IKAROS dimerization haploinsufficiency causes hematologic cytopenias and malignancies. Blood. (2021) 137:349–63. 10.1182/blood.2020007292 32845957 PMC7819759

[60] Thaventhiran JE Allen HL Burren OS Rae W Greene D Staples E. Whole-genome sequencing of a sporadic primary immunodeficiency cohort. Nature. (2020) 583:90–5. 10.1038/s41586-020-2265-1 32499645 PMC7334047

[61] Kellner ES Krupski C Kuehn HS Rosenzweig SD Yoshida N Kojima S. Allogeneic hematopoietic stem cell transplant outcomes for patients with dominant negative IKZF1/IKAROS mutations. J Allergy Clin Immunol. (2019) 144:339–42. 10.1016/j.jaci.2019.03.025 30965037

[62] Ding Y Zhang B Payne JL Song C Ge Z Gowda C. Ikaros tumor suppressor function includes induction of active enhancers and super-enhancers along with pioneering activity. Leukemia. (2019) 33:2720–31. 10.1038/s41375-019-0474-0 31073152 PMC6842075

[63] Read KA Jones DM Freud AG Oestreich KJ. Established and emergent roles for Ikaros transcription factors in lymphoid cell development and function. Immunol Rev. (2020) 300:82–99. 10.1111/imr.12936 33331000 PMC8015388

[64] Cai Q Dierich A Oulad-Abdelghani M Chan S Kastner P. Helios deficiency has minimal impact on T cell development and function. J Immunol. (2009) 183:2303–11. 10.4049/jimmunol.0901407 19620299

[65] Inui M Miyado M Igarashi M Tamano M Kubo A Yamashita S. Rapid generation of mouse models with defined point mutations by the CRISPR/Cas9 system. Sci Rep. (2014) 4:1–8. 10.1038/srep05396 24953798 PMC4066261

[66] Birling M-C Herault Y Pavlovic G. Modeling human disease in rodents by CRISPR/Cas9 genome editing. Mamm Genome. (2017) 28:291–301. 10.1007/s00335-017-9703-x 28677007 PMC5569124

